# The WHO AFRO external quality assessment programme (EQAP): Linking laboratory networks through EQA programmes

**DOI:** 10.4102/ajlm.v5i2.560

**Published:** 2016-10-17

**Authors:** Debrah I. Boeras, Rosanna W. Peeling, Philip Onyebujoh, Ali A. Yahaya, Hieronyma N. Gumede-Moeletsi, Jean B. Ndihokubwayo

**Affiliations:** 1 London School of Hygiene and Tropical Medicine, London, United Kingdom; 2 World Health Organization Regional Office for Africa, Brazzaville, Republic of Congo

## Abstract

External Quality Assessment (EQA) surveys performed by the World Health Organization Regional Office for Africa (WHO AFRO) revealed the need for the strengthening of public health microbiology laboratories, particularly for testing of epidemic-prone diseases in the African Region. These surveys revealed common issues such as supply chain management, skilled personnel, logistical support and overall lack of quality standards. For sustainable improvements to health systems as well as global health security, deficiencies identified need to be actively corrected through robust quality assurance programmes and implementation of laboratory quality management systems.

Given all the pathogens of public health importance, an external quality assessment programme with a focus on vaccine-preventable diseases and emerging and re-emerging dangerous pathogens is important, and should not be stand-alone, but integrated within laboratory networks as seen in polio, measles, yellow fever and rubella.

In 2015, WHO AFRO collaborated with the US Centers for Disease Control and Prevention, the London School of Hygiene & Tropical Medicine and partners in a series of consultations with countries and national and regional EQA providers for the development of quality assurance models to support HIV point-of-care testing and monitoring. These consultations revealed similar challenges as seen in the WHO AFRO surveys. WHO AFRO brought forth its experience in implementing quality standards for health programmes, and also opened discussions on how lessons learned through such established programmes can be utilised to supporting and strengthening the introduction of early infant diagnosis of HIV and viral load point-of-care testing.

An optimised external quality assessment programme will impact the ability of countries to meet core capacities, providing improved quality management systems, improving the confidence of diagnostic network services in Africa, and including capacities to detect events of international public health importance.

An external quality assurance programme (EQAP) is critical for laboratories to provide high-quality test results. The EQAP encompasses: (1) investments in building human capacity; (2) investments in building laboratory management systems, infrastructure and management of quality systems; (3) well-written policies and procedures; (4) a quality control system, quality improvement (QI) and external quality assessment (EQA); and (5) accreditation standards^1^.

Laboratory professionals should routinely perform quality control testing to guarantee that test methods and equipment perform according to established standards. Laboratory professionals must participate in EQA/proficiency testing programmes in order to demonstrate that acceptable systems are in place and that specimens are collected and processed appropriately.

The objectives of the World Health Organization Regional Office for Africa (WHO AFRO) EQAP are:

- To support countries in their efforts to improve their laboratory quality management systems, including accreditation.
- To collect, analyse, and report laboratory EQA feedback data and use this information for corrective action and laboratory capacity strengthening.
- To establish recognised, accurate, and sustainable diagnostic practices using appropriate algorithms.

## World Health Organization Regional Office for Africa laboratory networks

**FIGURE 1:**
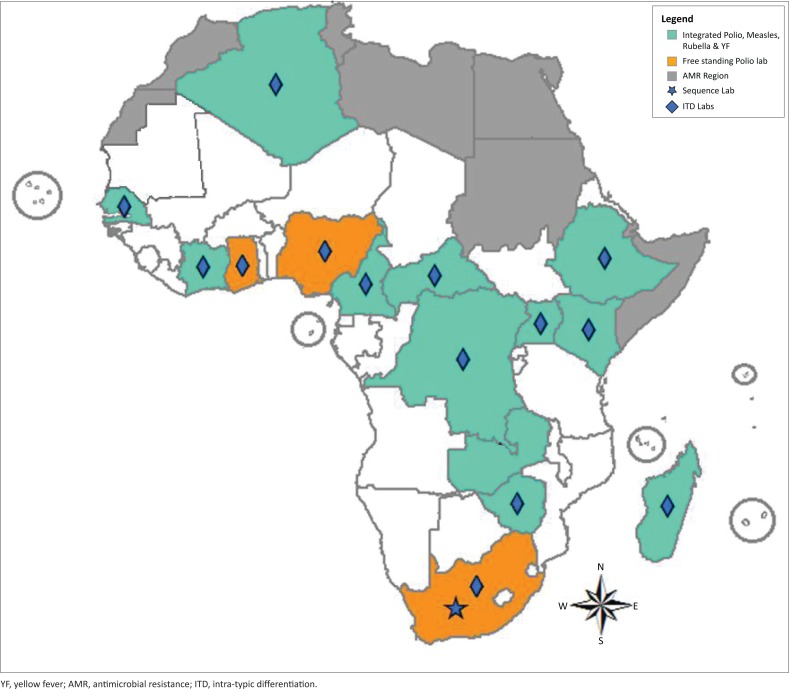
Vaccine-preventable disease networks in Africa.

WHO AFRO has established and monitored several national/regional laboratory networks linked by EQA programmes, including polio, measles and rubella, yellow fever, rotavirus and influenza laboratories, the Emerging and Dangerous Pathogens Laboratory Network, and the epidemic-prone bacterial diseases laboratory network, including tuberculosis. The Polio Laboratory Surveillance Network was established in Africa in 1993 to support the Global Polio Eradication Initiative. This laboratory network is made up of 16 laboratories in 15 countries, with each laboratory supporting at least one other country. Of these, 12 polio laboratories have integrated functions supporting measles, yellow fever and rotavirus programmes (Figure 1).

**FIGURE 2:**
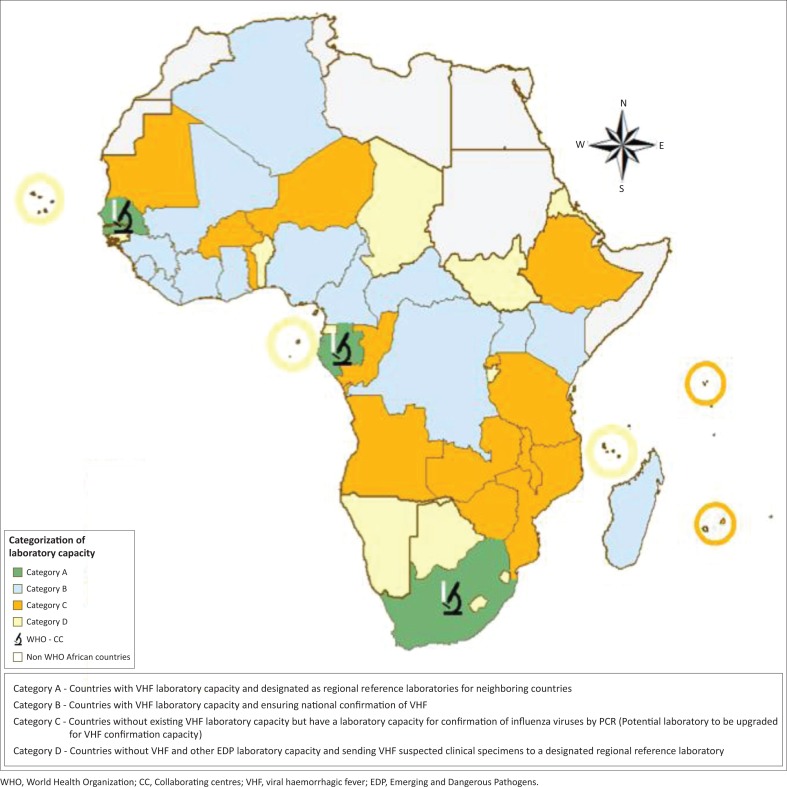
Emerging and Dangerous Pathogens Laboratory Network.

The Emerging and Dangerous Pathogens Laboratory Network has been established for the confirmation of viral haemorrhagic fevers in the WHO African region. The network consists of laboratories in 17 countries, categorised based on their capacity, as described in Figure 2.

**FIGURE 3:**
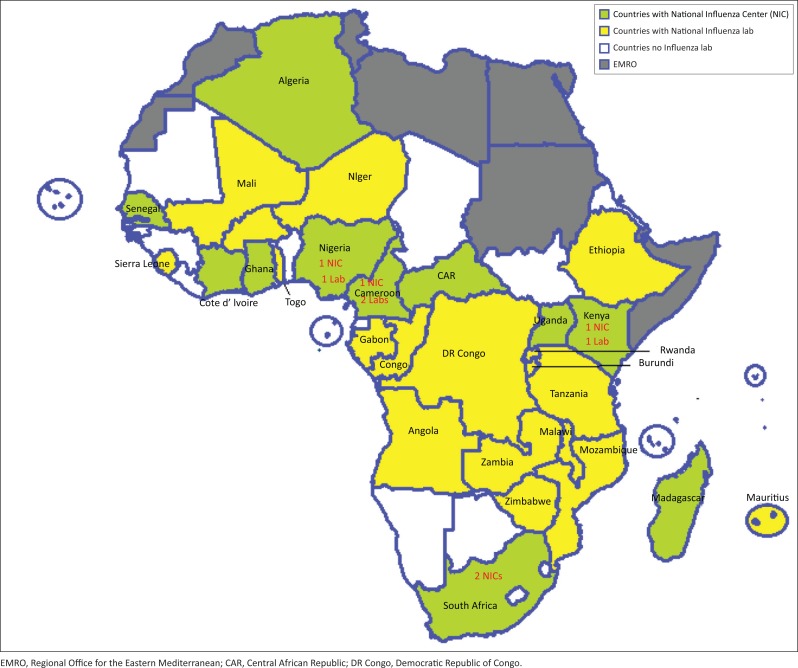
The World Health Organization African Region influenza laboratory network.

The laboratory capacity to diagnose influenza infections by real-time PCR in the African Region has grown since the 2009 pandemic. Presently, the regional influenza laboratory network comprises 30 national influenza reference laboratories in 30 countries. Currently, 14 countries are registered as National Influenza Centres and are members of the WHO Global Influenza Surveillance Network in the African Region (Figure 3). Since 2010, data on virological surveillance of the influenza virus have been received weekly from 22 countries. Presently, 17 countries have a functional national influenza-like illness and severe acute respiratory infections surveillance system. In 2015, more than 35 000 samples were tested, around 15% of which were positive. As of this writing, more than 21 000 samples have been tested, and more than 10% were positive. This kind of information aids in identifying seasonal patterns of influenza in the region and informing influenza vaccination to prioritise high-risk groups.

## External quality assessment programmes for the World Health Organization Regional Office for Africa

**FIGURE 4:**
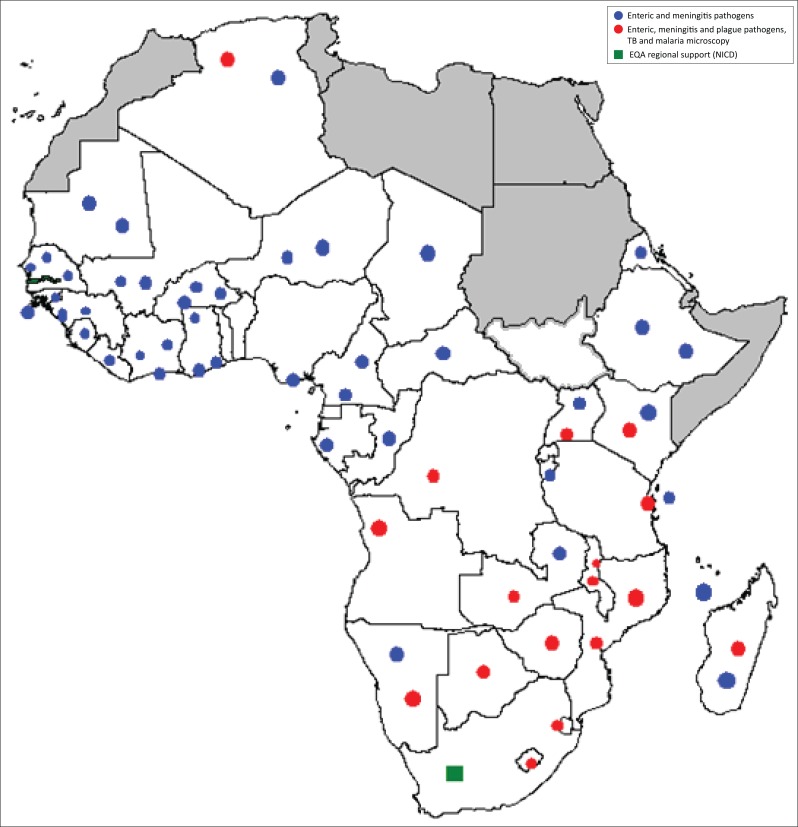
World Health Organization-National Institute for Communicable Diseases microbiology external quality assessment programme for potential bacterial epidemics, tuberculosis and malaria in the African region (since 2002).

WHO AFRO collaborated with the National Institute for Communicable Diseases (NICD) in South Africa as the EQA provider.^2^ The WHO/NICD microbiology EQAP was started in May 2002 and currently, over 90 laboratories in 45 African countries participate in this programme at a cost of USD 150 000/year (Figure 4).

Its objective is to assess laboratory capacity to detect epidemic-prone diseases (enteric and meningitis pathogens and plague). In addition, this programme assesses laboratory capacity to diagnose malaria and tuberculosis.^3^ The NICD has the capacity to produce, perform quality control of and distribute samples for the EQAP. The resulting data goes to both NICD and WHO AFRO, belonging to the WHO AFRO EQAP programme.

The EQAP conducts three surveys (shipments) per calendar year, with 2 to 4 disease components per survey:

- Bacteriology discipline: six simulated specimens containing organisms isolated from enteric, meningeal and routine clinical samples for microscopy, culture, identification and/or antimicrobial susceptibility testing.
- Plague discipline: two simulated samples for diagnostic tests for *Yersinia pestis*, including microscopy.
- Tuberculosis microscopy: 10 slides per panel for reporting on the presence of acid-fast bacilli using the International Union for Tuberculosis and Lung Disease smear-grading system.
- Malaria microscopy: 20 slides per panel to assess ability to diagnose malaria species and to count parasites.

Laboratories for enteric and meningitis pathogens were graded based on a score depending on their capacity to do the following:

1. Microscopy: clinically important, basic skill, relevant.
2. Culture and identification.
3. Serotyping/serogrouping: important to evaluate disease prevention and control strategies.
4. Antimicrobial agents selected for testing.
5. Antimicrobial susceptibility testing.

In the region, it was observed that errors in antimicrobial resistance testing included inappropriate selection of antibiotics, guideline availability, no adherence to guidelines, no control/reference strains, and incorrect results and interpretation.^4^ These surveys reflect common issues, such as shortages of supplies and human resources, as well as the critical need for national standards and quality assessments.^5^

## Diagnostic challenges for tuberculosis control

**FIGURE 5:**
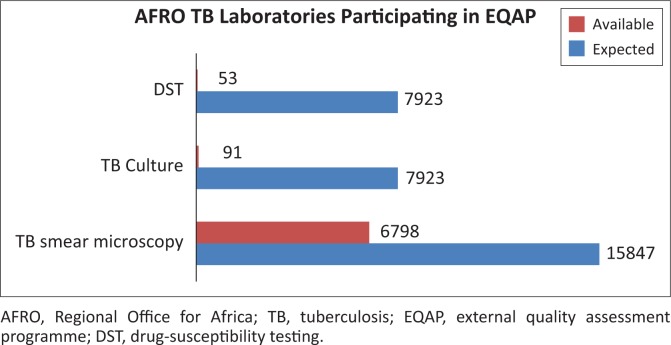
Facilities available in the African region for tuberculosis diagnostic testing subscribing to an external quality assessment programme.

As shown in Figure 5, there are a total of 15 847 laboratories in the region that have the capacity to perform tuberculosis smear microscopy, with less than half (6798) subscribing to an EQA programme. Of the 7923 laboratories that can perform tuberculosis culture and drug-susceptibility testing, only 91 subscribed to an EQA programme for culture and 53 for drug-susceptibility testing services. It also highlights the enormous challenges inherent in meeting the WHO ‘End TB’ Strategy which advocates for universal drug-susceptibility testing coverage. Now, instead of performing cultures, many facilities are moving to molecular testing, the quality of which can be assured through a fee-based National Health Laboratory Services tuberculosis programme for the Cepheid GeneXpert^®^.^6^

## External quality assessment programme corrective action

As part of EQA, laboratories with low performance in the EQAP receive feedback, training and technical support aimed at instituting corrective action. In a joint collaboration, the EQAP and NICD provide feedback and corrective action to all participating laboratories in the three official working languages in the WHO African Region (English, French and Portuguese). On-site and regional training sessions are also provided. As part of technical support, standard operating procedures and guidelines are made available. Specifically, guidelines and standard operating procedures for laboratories performing antimicrobial susceptibility testing are made available in addition to laboratory bench training workshops.

## Way forward

In 2015, WHO AFRO collaborated with the US Centers for Disease Control and Prevention, the London School of Hygiene & Tropical Medicine and partners in a series of consultations with countries and international, national and regional EQA providers for the development of quality assurance models to support HIV point-of-care testing. Participants consulted revealed similar challenges with supply chain management, skilled personnel, logistical support and overall lack of quality standards. WHO AFRO provided experiential oversight in implementing quality standards for health programmes, as well as facilitating discussions on how lessons learned through such established programmes should be utilised in supporting and strengthening the introduction of HIV early infant diagnosis and viral load point-of-care testing.

## Conclusion

The EQAP has provided a clearer understanding of the capacities of participating laboratories to produce accurate results. This international EQAP provides a unique opportunity for African national public health laboratories to continuously assess and improve their laboratory performance, identifying their strengths, limitations and on-going challenges. The EQAP facilitates an improvement in the confidence of test results from diagnostic networks that impacts on treatment capacities by the requesting treatment centres.

Given all the pathogens of public health importance, the EQAP should not be stand-alone, but rather integrated within laboratory networks, as seen with polio, measles, yellow fever and rubella. Grouping them together provides economy of scale for cost-effectiveness and efficiencies for ease of training and reporting. The EQAP needs to emphasise the utility of such programmes in improving services for both vaccine-preventable diseases and emerging and re-emerging dangerous pathogens.

Finally, an optimised EQAP will impact the ability of countries to meet International Health Regulations (2005) core capacities, including the capacity to detect events of international public health importance. This programme has allowed WHO AFRO to identify the capacities and resources needed by countries for sustainable improvement and competency of diagnostic services throughout Africa. Improved quality of laboratory networks produces highly-accurate surveillance data which is required to estimate disease burden in countries, identify outbreaks and inform strategies for disease control and prevention.
